# Determination of the Levels of Polycyclic Aromatic Hydrocarbons in Toasted Bread Using Gas Chromatography Mass Spectrometry

**DOI:** 10.1155/2010/821216

**Published:** 2010-08-24

**Authors:** Amal Al-Rashdan, Murad I. H. Helaleh, A. Nisar, A. Ibtisam, Zainab Al-Ballam

**Affiliations:** Chromatographic Section, Central Analytical Chromatography (CAL), Kuwait Institute for Scientific Research (KISR), P.O. Box 24885, 13109 Safat, Kuwait

## Abstract

Concentration of 16 polycyclic aromatic hydrocarbons (PAHs) in eighteen baked bread samples using gas oven toasting were evaluated in this study. Samples were classified into the following categories: (1) bread baked from white wheat flour, (2) bread baked from brown wheat flour, and (3) sandwich bread baked from white wheat flour. Analysis was performed by GC-MS after Soxhlet extraction of the sample and clean up of the extract. The levels of B[a]P was not detected in ten of eighteen samples. In the rest of the samples, B[a]P are varied from 2.83 to 16.54 *μ*g/kg. B[a]A, CHR, B[b]FA, B[k] FA, IP, DB[a,h]A, and B[ghi]P concentrations were found to be less than 10.0 *μ*g/kg. However, B[a]P are not detected in original white and brown wheat flour. The total PAHs were varied in the range 1.06–44.24 *μ*g/kg and 3.08–278.66 *μ*g/kg for H-PAH and L-PAH, respectively. Reproducibility and repeatability of the proposed method was calculated and presented in terms of recovery and relative standard deviations (RSD, %). Recoveries were varied from 72.46% to 99.06% with RSD ± 0.28–15.01% and from 82.39% to 95.01% with RSD ±1.91–13.01% for repeatability and reproducibility, respectively. Different commercialized samples of toasted bread were collected and analyzed.

## 1. Introduction

Polycyclic aromatic hydrocarbons (PAHs) are a group of chemicals having aromatic ring. Those containing up to four benzene rings are known as light PAHs (L-PAHs) and those containing more than four benzene rings are known as heavy PAHs (H-PAHs). H-PAH are more stable and toxic than L-PAHs [1]. Some studies have shown that heavy PAHs can induce dioxin-like activity and weakened estrogenic responses [2]. Most PAHs are formed from incomplete combustion of wood, oil, coal, and garbage [3]. Some have been demonstrated to be mutagenic and carcinogenic for humans [4]. Those PAHs that have not been found to be carcinogenic may, however, synergistically increase the carcinogenicity of other PAHs [5, 6]. Human exposure to PAHs does not occur singly, it encountered in complex mixtures of PAHs [7]. The main source of human exposure to PAHs is to be through diet with grains and vegetables, being the major dietary sources [5].

Foodstuffs can be contaminated by PAHs in several ways, such as either direct or indirect contact with smoke. The routes through which PAHs travel include food, air, and water [8]. The highest levels of PAHs are found in foodstuffs, both processed and unprocessed [9]. PAHs have been detected in seafood, beverages, smoked foods, and broiled meat [10]. 

Several methods for the analysis of B[a]P in food have been published. The first method, published in the 1970s, was based on ultraviolet (UV) absorption of an extract, using high-performance liquid chromatography with fluorescence detection (HPLC-FLD) [11]. Germans published two methods for the screening of B[a]P levels in smoked and nonsmoked meat products [12]. These methods were extended to be used with general foods in 2004.

European Union legislation (EUL) sets a maximum allowed concentration for benzo[a]pyrene B[a]P in different food product in the range 1–10 *μ*g/kg [13], and for benzo[a]anthracene (B[a]A) and Benzo[a]pyrene (B[a]P) in liquid smoke flavoring of 20 *μ*g/kg [14]. B[a]P recommended allowable daily intakes range from 0.04 to 0.42 *μ*g/day in Italy [15], whereas in Spain, the daily recommended allowable intakes for B[a]P and total PAHs was 0.14 and 8.6 *μ*g/day, respectively, for bread, cookies, cakes, rice, and so forth. [16].

Baked and packaged bread consumption has increased dramatically in most of the countries, yet no regulations of the maximum allowable levels for PAHs in bread have been established. Rey-Salgueiro et al. [17] have tested the effects of toasting procedures on the polycyclic aromatic hydrocarbons (PAHs) levels in bread, and they reported that PAH levels are up to 350 *μ*g/kg in toasted samples when using a wood flame. Determination of the 16 PAHs in nonfatty foods (i.e., mashed potato, potato, and toasted bread) showed total PAH levels in the toasted bread samples in the range 7.38–18.0 *μ*g/kg and in Ortiz toasted bread samples the levels of chrysene were equal to 7.38 *μ*g/kg [18]. Scientists reported the concentration of some PAHs and heavy metals in baking bread when using different fuel. Average PAHs levels of 320.6, 158.4, 317.3, and 25.5 *μ*g/kg have been reported for baked bread using mazot, solar, solid waste, and electricity, respectively [19]. 

Super critical fluid extraction and HPLC with fluorimetric detection have been developed to determine some selected PAHs in toasted bread. PAHs have been found in the range 0.323–9.40 *μ*g/kg with this method [20].

Recently, PAHs in baked bread using wood as the fuel to estimate the total concentration of the 16 PAHs were varied from 6 to 230 *μ*g/kg dry weight (d.w.) [21].

European Union has stressed and recommended that PAHs to be measured in as wide as possible in food products in order to obtain data on the occurrence and specific concentrations in a variety of matrices [22]. Yet, there remains a general lack of information on the levels of PAHs in different type of breads. To our knowledge, there are no reports or information on the occurrence of PAHs in certain species in Kuwait.

Bread is a good source of energy and it contains minerals, proteins, vitamins, and lipids which are considered to be essential for human nutrition. In Kuwait, bread is produced by the Kuwait Flours Mills and Bakeries Company, as well as by a number of smaller bakeries. In most of the bakeries in Kuwait, the gas oven toasting is the main source of energy utilized for processing.

Wheat is considered to be a major component of foods for humans [23]. Wheat flours used in making bread is important. Although, Tuominen et al. and Dennies et al. [24, 25] have reported that PAHs are present in wheat flour. 

This paper aims to give a brief overview on the current levels of PAHs in food, especially in bread samples of white and brown wheat flours used in baking bread in Kuwait.

There is a strong believe that there is a gap, in the information available on PAHs levels in toasted bread made from brown and white flour. An experiment was developed to investigate the 16 PAHs in toasted bread. Finally several commercial bread samples were analyzed in order to establish maximum ranges of PAHs in bread and in flour.

## 2. Experimental

### 2.1. Standard and Calibration Solution

Target standard compounds (PAH mixture Quebec, Ministry of Environment, Cat.No. 502065, PA, USA) included: NA, ACL, AC, FL, PHE, AN, FA, PY, B[a]A, CHR, B[b]FA, B[k]FA, B[a]P, IP, DB[ah]A, and B[ghi]P. A mixture of deuterated PAHs (d-PAHs), namely, acenaphthalene-d10, phenanthrene-d10, chrysene-d12, and perylene-d12 (2000 *μ*g/ml, Catalog No. 8500-6076) (obtained from Agilent, Foster City, CA, USA) were used to prepare an internal standard solution (1 ng/*μ*l) to quantitate the relative native PAHs.

PAHs were prepared by dissolving about 0.01 g in hexane. From these solutions, mixed solution of PAHs ranging from 100–1000 pg/*μ*l were prepared in hexane. These solutions were stored in amber flasks at 4°C. Three PAHs calibration points were prepared: 100, 500, and 1000 pg/*μ*l. d-PAHs (1000 pg/*μ*l) were added to stock calibration solutions as well as to the samples before extraction. The samples were corrected for recoveries by applying an internal standard method, since PAHs and d-PAHs follows the similar steps in extraction and analysis.

### 2.2. Chemicals and Materials

All solvents were of HPLC-grade. Hexane and dichloromethane were supplied by Merck (Darmstadt, Germany). The chromatographic column was used for clean up of the extract, filled with 2 g of silica gel and 1 g of aluminium oxide. Nitrogen gas was used to concentrate the extract. Evaporator (Heidolph-Verwenden, Germany), 100–200 mesh silica gel (Aldrich, Steinhein, USA), anhydrous sodium sulfate (EMD-Chemical, Darmstadt, Germany) and 70–230 mesh aluminium oxide were purchased from Sigma in Steinhein-Germany.

### 2.3. Extraction Procedure and Sample Preparation

All bread and flour samples (5 g) were spiked with d-PAHs, and extracted with mixed solvents of 150 ml hexane : dichloromethane (1 : 1), using a Soxhlet apparatus for 16 h. The solvents were reduced to 1 ml using a rotary evaporator and N_2_ gas. The extract was then passed through a clean up column filled with 2 g of 100–200 mesh silica gel, followed by 1 g of aluminum oxide (considered to be the most efficient and selective for column chromatography separation) and anhydrous sodium sulfate. The column was washed with 10 ml of hexane and the PAHs were collected by eluting the column with 8 ml of hexane and 5 ml of dichloromethane. Finally, the extract was concentrated to 1 ml under a weak nitrogen flow at ambient temperature. This solution is then injected to GC/MS for PAHs analysis. Quantification of individual PAHs was based on internal calibration standard containing known concentrations of 16 PAHs and d-PAHs.

### 2.4. Samples

Bread samples were collected from more than 8 different bakeries located in Kuwait City. The bread samples were ground, threshed, and stored in amber glass bottles with Teflon-lined caps at −20°C until extraction. For a reference, 5 g of each bread and flour was subjected to Soxhlet solvent extraction. The bread description and their ingredients content are listed in Table 1.

### 2.5. Gas Chromatography—Mass Spectrometry Conditions

PAH analysis was conducted using an Agilent 5890, Series II, gas chromatography (Agilent, Avondale, USA) interfaced to a mass selective detector (Agilent 5972, Agilent, Avondale, USA). Separation of PAHs were performed using a 5% phenyl-methylsilicone (DB-5MS) bonded-phase fused-silica capillary column (Hewlett-Packard, 30 m × 0.25 mm i.d., film thickness 0.25 *μ*m). The injector port was run in splitless mode. The oven temperature program was 45°C for 2 min, raised to 290°C at a rate of 10°C/min and maintained at this temperature for 8 min. The transfer line was maintain at 295°C. The mass spectra were collected by electronic impact at 70 eV. Stock solutions were used to establish the retention time of each analyte. Detection of PAHs was carried out using SIM mode, which is designed for preselected ion peaks, nonselected peaks are not identified and quantified.

## 3. Results and Discussion

### 3.1. GC-MS Procedure Performance

**Figure 1:**
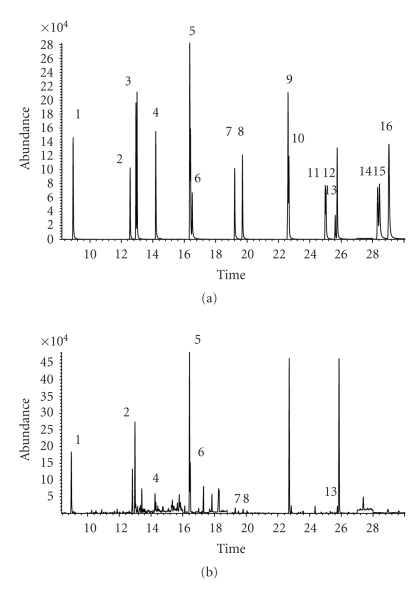
(a) and (b) Chromatogram of polycyclic aromatic hydrocarbons (PAHs) obtained using the GC-MS method. Numbers on the chromatogram refer to the individual PAHs as indicated in Table 2. (a) Standard PAHs (500 pg/*μ*l), (b) Bread sample.

The chromatograms of the 16 selected PAHs in the standards and bread samples are shown in Figures 1(a) and 1(b). The PAHs shows a wide spectrum of volatility and all 16 PAHs behave the same in chromatographic area in the standard and the sample. The set of samples was analyzed along with a blank for PAH background correction. The results were corrected for recoveries using an internal standard method, assuming that PAHs and d-PAHs behave in a similar manner during extraction and analysis. Recoveries of d-PAHs were utilized to estimate recoveries of the native PAHs. The average recoveries of d-PAHs varied from 79.11% to 91.56% with relative standard deviation (RSDs) ranging from 7.02% to 8.86%.

### 3.2. Linearity of the Calibration Curve

The linearity of the method for the analysis of PAHs was evaluated by building the calibration curves for the obtained chromatographic area versus the concentration of PAHs, ranging from 100–1000 pg/*μ*l for each PAH. The correlation coefficient for most of the PAHs is acceptable since it is above 0.995, which is acceptable. Table 2, summarizes the analytical characteristics of the method with regards to PAHs standard.

### 3.3. Repeatability, Reproducibility, and Recovery

Blank sample of bread were spiked with different concentrations of each PAH. Reproducibility was evaluated by analyzing one sample on 3 different days. Repeatability was performed by running two analyses on the same day under the same conditions. Results are presented in Table 3. Recoveries varied between 81.96% and 95.01%, with RSDs between 1.91% and 13.01% for reproducibility. Recoveries were varied between 72.46% and 99.06%, with RSDs between 0.28% and 15.0% for repeatability. The results obtained shows that the method is very accurate and precise.

### 3.4. Limit of Detection (LOD) and Limit of Quantification (LOQ)

The limit of detection (LOD) is defined as the lowest concentration leading to a signal-to-noise ratio of 3 whereas the limit of quantification (LOQ) is defined as the concentration leading to a signal-to-noise ratio of 10 (Table 4). A set of seven analytical blanks were analyzed. LODs were calculated for any PAH detected. LOD is calculated at three standard deviation of the blank. The LOD for the PAHs not found in the blanks were based on the instrument detection limits for the lowest standard level. The signal-to-noise ratio for each PAH peak was calculated in comparison to a baseline section close to the peak. Instrumental LODs and LOQs were evaluated using a standard comprise of 16 mixture of the PAHs mixture. 

### 3.5. Matrix Effects

The matrix effect was evaluated by spiking the bread samples with a concentration range used for calibration, then comparing the correlation coefficient and the slope of spiked standard to the matrix with the original standard calibration curves. No matrix effect was observed for any of the PAHs in any type of the bread matrices.

### 3.6. Determination of PAHs in Bread Samples

The results of bread toasted by electric oven toasting showed that, no significant increases in PAH levels, with regard to H-PAH and L-PAH, even when the bread was toasted at higher temperature. In our study a soft bread baked from white wheat flour toasted at three different temperatures, such as 250°C, 350°C and 500°C for 20 min, no PAHs were detected. However, gas oven toasting of bread is the general practice utilized in most of the bakeries allocated in Kuwait. Therefore, all of the bread tested in this study were based on gas oven toasting.

In order to evaluate the PAHs in toasted bread, most of the bread were commercially collected. In contrast, the original wheat flour of white and brown, which is normally used in baking the bread were evaluated. The results shows that the brown wheat flour was completely free from H-PAH. The presence of FA and PY was observed in the range of 1.19–2.19 and 0.71–1.66 *μ*g/kg for brown and white wheat four, respectively. Some L-PAH was detected, such as NA, FL, and PHE with a concentration of 4.33 *μ*g/kg, 5.04 *μ*g/kg, and 31.4 *μ*g/kg for white wheat flour and of 12.9 *μ*g/kg, 8.14 *μ*g/kg, and 32.0 *μ*g/kg for brown wheat flour, respectively.

In this study, the commercially toasted bread samples were collected from the local market and classified according to the type of wheat flour used in baking and they are as follows.

(1) Bread baked from white wheat flour, (2) bread baked from brown wheat flour, and (3) sandwich bread baked from white wheat flour.

**Figure 2:**
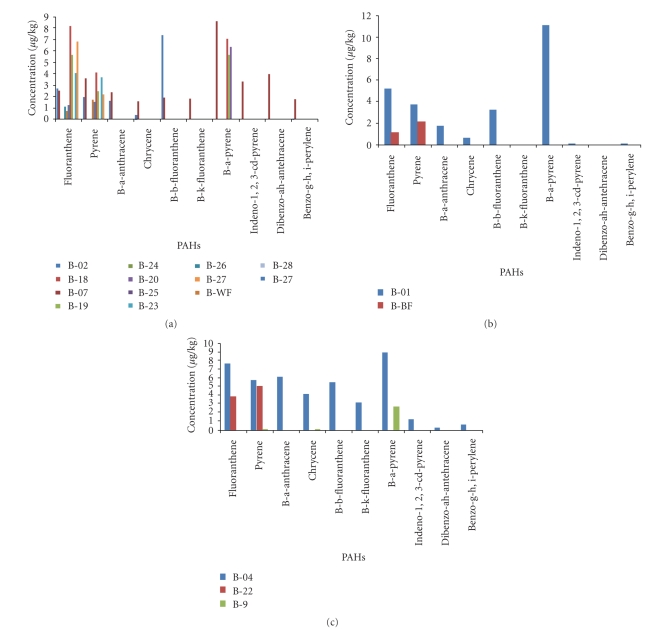
(a) Concentrations of PAHs obtained from bread samples baked with white Wheat flour (b) Concentrations of PAHs obtained from bread samples baked with Brown Wheat flour (c) Concentration of PAHs obtained from sandwich bread sample baked with white flour.

Figure 2(a), shows the bread result obtained from white wheat flour baking. B[a]P was detected in 8 samples of the 18 samples in the range of 2.83–16.54 *μ*g/kg. L-PAH were ranged from 3.91 to 278.66 *μ*g/kg and H-PAH were ranged from 0.06 to 22.32 *μ*g/kg. The total PAHs (∑PAH) for the 18 tested samples were found in the levels of 1.06–287.5 *μ*g/kg.

In samples of bread baked using white wheat flour, B[a]P was not detected in most of the samples. Five samples were exceeded the legal permissible limits (1 *μ*g/kg) proposed by the European Union for processed cereal-based foods [26]. Dennis et al. [27] reported the levels of B[a]P in white flour was found to be less than 0.1 *μ*g/kg, although they reported 2.2 *μ*g/kg in cereal-derived products enriched with high levels of edible oils. In our investigation, the original white wheat flour was analyzed in parallel to the toasted white bread, B[a]P was not detected in white flour, supplied by the Kuwait Flour Mills and Bakeries Company.

Figure 2(b), shows the brown wheat bread result. B[a]P was detected in one sample and found to be equal to 11.13 *μ*g/kg. 

Sandwich bread baked from white wheat flour showed in Figure 2(c). The levels of B[a]P was in the range of 2.83–9.04 *μ*g/kg in 2 of the 3 samples.

Table 5 summarizes the levels of higher molecular weight and lower molecular weight PAHs that were found in each type of tested sample. 

Owing to our knowledge, there are few publications for PAHs in toasted bread [17, 18, 20]. Rey-salgueiro et al. [17], detected out of 24 selected samples were in the range of 0.25–0.45 *μ*g/kg, 0.006–0.098 *μ*g/kg, and 0.13–0.23 *μ*g/kg for B[b] F, B[k] F and B[a]P, respectively. In another two samples of the 24 samples, B[a]A were 0.16 and 0.19 *μ*g/kg. PAHs levels were found in the samples analyzed by Kayali-sayadi et al. [20], were varied from 0.32 to 9.4 *μ*g/kg in toasted bread for the following PAHs: NA, AC, PHE and DB[ah]A. Nieva-Cano et al., detected the following PAHs: FL = 9.52–12.1 *μ*g/kg, PHE = 15.4 *μ*g/kg, AN = 7.38–11.7 *μ*g/kg, FA = 17.8–18.0 *μ*g/kg, and CHR = 9.73 *μ*g/kg in three types of the bread. 

No legal limit set for most of the European Union (EU), but some countries have set maximum levels [22]. Ahmed et al. [19] have studied the total concentration of PAHs in bread, an average of 320.6, 158.4, 317.3, 25.5 *μ*g/kg were found, using mazot, solar, solid waste, and electricity, respectively. The total concentration of the 16 PAHs detected by Orecchio and Papuzza were found to vary from 6 to 230 *μ*g/kg (d.w.) in baked bread [21].

The results obtained in the current study showed a distinguish distribution of single PAH in the toasted bread samples. This contribution may refer to the baking method used by the baker in toasting the bread or on the temperature variation of the gas oven.

NA, FL, and PHE are the most three abundant compounds found in the tested bread sample. Naphthalene (NA) and Phenanthrene (PHE) were abundant than other PAHs. When wood were used as a fuel, concentration of B[a]P was ranged from 0.13 to 9.4 *μ*g/kg. Eight samples exceeds the permissible limit of 1 *μ*g/kg proposed for processed cereal-based foods [26].

Total levels of the 16 PAHs in the samples analyzed by the proposed method (i.e., 257.3 and 1114 *μ*g/kg) were higher than those reported by Ahmed et al., when electricity and solar power were used for baking bread, but were lower (i.e., 320.6 and 317.3 *μ*g/kg) than those obtained when mazot and solid waste were used as fuel [19]. The obtained values in the current study were also higher than those obtained by Orecchio and Papuzza [21] when wood was used as fuel.

The results obtained in this study showed that some selected bread samples have higher concentration of PAHs, mainly those of H-PAH, above the maximum limits compared to other type of food. Some of the so-called Iranian bread tested in this study, have high levels of PHE and NA, this could be due to the baked bread is subjected directly to the high flame of gas, which leads to bake the bread in a short time.

B[a]P equivalent was calculated to indicate the carcinogenic of PAHs in the sample using equation reported by Brown and Mittelman [28], it was found to be in the range of 0.06 to 24.0 *μ*g/kg. The obtained value in this study were found to be higher than the existing methods [19, 21].

The daily intakes of B[a]P, based on consumption of 300 g of bread per day, were found to be in the range of 0.85–4.96 *μ*g/day/person. The obtained values were comparatively lower than those reported by Orecchio and Papuzza [21] and Ahmed et al., [19].

The presence of PAHs in food is significantly due to heat processes such as smoking, smoke-drying, and grilling. However, environmental pollutants are also considered to be an issue [29]. Moret et al. conform the facts when they studied some types of edible oils (e.g., olive oil) may be contaminated with PAHs, due to artificial drying and heating during processing [30, 31].

Our results showed that the PAHs detected in bread samples were originated from gas oven toasting.

## 4. Conclusions

As far as to our knowledge, there have been no publications on the determination of PAHs in different types of bread baked from white and brown wheat flour and nonbaked flour.

The method proposed and tested in this study provided a database that is urgently in need for this area. The levels obtained in this study were comparable to other methods except for some selected bread samples which were higher than maximum levels.

## Figures and Tables

**Table 1:** Commercial toasting breads and flours samples.

Sample Code	Principal ingredients
B-01	Brown bread
B-02	Soft Lebanese bread baked from white flour
B-04	Sandwich baked from white flour
B-07	Toasted Lebanese bread
B-09	French sandwich obtained from Al-Omara'a bakery
B-17	Iranian Bread baked from white flour—Salwa-A-11
B-18	Iranian Bread baked from white flour—Subah Al-Salem
B-19	Iranian Bread baked from white flour—Al-Qurean
B-20	Iranian Bread baked from white flour—Al-Khaldiyah
B-22	French Sandwich obtained from Delice De France.
B-23	Iranian Bread baked from white flour—Salwa-A-7
B-24	Toasted Lebanese bread at Temp. = 250°C, for 20 min
B-25	Toasted Lebanese bread at Temp. = 350°C, for 20 min
B-26	Toasted Lebanese bread at Temp. = 500°C, for 20 min
B-27	Iranian Bread baked from white flour—Kifan
B-28	Iranian Bread baked from white flour—Dasma
B-BF	Whole wheat flour (brown flour)
B-WF	White flour

**Table 2:** Calibration characteristics of the 16 PAHs.

No	PAHs	Correl. Coeff. (*R* ^2^)	Calibration range (pg/*μ*l)	*R*_*t*_ (min)	*m*/*z*	Reproducibility of the calibration curve (*n* = 4)		
						100 (pg/*μ*l)	500 (pg/*μ*l)	1000 (pg/*μ*l)
						Rec. ± RSD (%)	Rec. ± RSD (%)	Rec. ± RSD (%)
1	Naphthalene (NA)	1.000	100–1000	8.957	128/130	106.46 ± 2.12	108.98 ± 9.52	97.63 ± 2.81
2	Acenaphthylene (ACL)	0.985	100–1000	12.599	152/154	92.88 ± 0.71	108.77 ± 9.31	97.86 ± 2.53
3	Acenaphthene (AC)	0.999	100–1000	13.040	153/154	105.79 ± 0.39	105.95 ± 6.48	98.52 ± 1.74
4	Fluorine (FL)	0.997	100–1000	14.236	166/168	98.90 ± 0.78	106.71 ± 7.26	98.33 ± 1.96
5	Phenanthrene (PHE)	0.992	100–1000	16.457	176/178	114.78 ± 1.76	108.29 ± 8.89	97.93 ± 2.45
6	Anthracene (AN)	0.990	100–1000	16.561	176/178	80.06 ± 1.65	107.79 ± 8.41	98.16 ± 2.18
7	Fluoranthene (FA)	0.986	100–1000	19.276	200/202	83.85 ± 1.63	108.69 ± 9.29	97.87 ± 2.52
8	Pyrene (PY)	0.988	100–1000	19.773	200/202	83.18 ± 1.76	107.68 ± 8.29	98.13 ± 2.21
9	Benz [a] anthracene (BaA)	0.991	100–1000	22.651	226/228	101.04 ± 1.80	109.32 ± 9.85	97.79 ± 2.60
10	Chrycene (CHR)	0.988	100–1000	22.741	226/228	113.08 ± 0.56	109.25 ± 9.77	97.69 ± 2.74
11	Benzo[b] fluoranthene (BbFA)	0.990	100–1000	25.057	250/252	73.54 ± 5.44	109.39 ± 9.94	97.82 ± 2.58
12	Benzo[k] fluoranthene (BkFA)	0.990	100–1000	25.106	250/252	80.55 ± 6.06	109.34 ± 9.88	97.87 ± 2.51
13	Benzo[a]Pyrene (BaP)	0.991	100–1000	25.697	250/252	107.62 ± 4.52	108.59 ± 9.14	98.16 ± 2.17
14	Indeno[1,2,3-cd] Pyrene (IP)	1.000	100–1000	28.449	276/278	108.90 ± 6.16	107.45 ± 8.02	98.42 ± 1.84
15	Dibenzo[ah]anthracene (DBahA)	1.000	100–1000	28.562	276/278	101.71 ± 9.79	108.77 ± 9.34	98.09 ± 2.24
16	Benzo[ghi]perylene (BghiP)	0.999	100–1000	29.180	276/278	113.14 ± 4.45	107.33 ± 7.89	98.28 ± 2.02

Correl. coeff: correlation coefficient; *R*_*t*_: retention time; *m*/*z*: mass/ion ratio; Rec.: recovery; RSD: relative standard deviations.

**Table 3:** Validation of the analysis of the 16 PAHs applying the recommended method.

PAHs	Repeatability		Reproducibility	
	(*n* = 2 × 1)		(*n* = 1 × 3)	
	(100,1000 *μ*g/kg)		(500, 50 *μ*g/kg)	
	Recovery (%)	RSD (%)	Recovery (%)	RSD (%)
Naphthalene (NA)	89.58	10.22	89.84	10.02
Acenaphthylene (ACL)	84.65	7.19	88.05	9.94
Acenaphthene (AC)	97.15	1.36	92.64	10.97
Fluorine (FL)	90.99	8.83	88.28	7.29
Phenanthrene (PHE)	72.46	14.10	83.45	13.01
Anthracene (AN)	83.25	7.47	81.96	3.68
Fluoranthene (FA)	95.62	4.32	93.09	7.53
Pyrene (PY)	85.50	15.01	91.01	8.30
Benz [a] anthracene (BaA)	90.46	1.23	85.75	10.78
Chrycene (CHR)	99.06	11.96	89.41	2.97
Benzo[b] fluoranthene (BbFA)	91.53	0.31	87.04	1.91
Benzo[k] fluoranthene (BkFA)	88.63	4.95	83.62	3.19
Benzo[a]Pyrene (BaP)	80.52	8.96	82.39	4.99
Indeno[1,2,3-cd] Pyrene (IP)	89.31	9.82	86.38	8.99
Dibenzo[ah]anthracene (DBahA)	95.76	0.48	91.97	7.19
Benzo[ghi]perylene (BghiP)	91.40	0.28	95.01	8.92

*n*: number of measurement.

**Table 4:** Method and instrumental limit of detection (LOD) and limit of quantification (LOQ) of the 16 PAHs.

PAHs	Method		Instrument	
	LOD (*μ*g/kg)	LOQ (*μ*g/kg)	LOD (*μ*g/kg)	LOQ (*μ*g/kg)
Naphthalene (NA)	0.6	1.8	3.0	9.0
Acenaphthylene (ACL)	0.8	2.4	4.0	12
Acenaphthene (AC)	0.8	2.4	4.0	12
Fluorine (FL)	1.0	3.0	5.0	15
Phenanthrene (PHE)	0.9	2.7	4.5	13.5
Anthracene (AN)	0.4	1.2	2.0	6.0
Fluoranthene (FA)	0.5	1.5	2.5	7.5
Pyrene (PY)	1.0	3.0	5.0	15
Benz [a] anthracene (BaA)	0.1	0.3	0.5	1.5
Chrycene (CHR)	0.1	0.3	0.5	1.5
Benzo[b] fluoranthene (BbFA)	0.2	0.6	1.0	3.0
Benzo[k] fluoranthene (BkFA)	0.1	0.3	0.5	1.5
Benzo[a]Pyrene (BaP)	0.1	0.3	0.5	1.5
Indeno[1,2,3-cd] Pyrene (IP)	0.2	0.6	1.0	3.0
Dibenzo[ah]anthracene (DBahA)	0.1	0.3	0.5	1.5
Benzo[ghi]perylene (BghiP)	0.3	0.9	1.5	4.5

**Table 5:** Levels of PAHs in bread and flours samples (ng/g), concentration are reported based on duplicate measurements.

PAHs	B-02	B-07	B-24	B-25	B-26	B-WF	B-17	B-18	B-19	B-20	B-23	B-27	B-28	B-01	B-BF	B-04	B-22	B-9
NA	ND	ND	12.9	12.0	ND	4.33	28.9	ND	ND	22.0	ND	177	45.9	3.08	12.9	4.79	1.00	61.6
ACL	ND	ND	ND	ND	ND	ND	ND	2.50	ND	3.38	ND	5.55	ND	ND	ND	ND	ND	ND
AC	ND	ND	ND	ND	ND	ND	ND	ND	ND	ND	ND	ND	ND	ND	ND	ND	ND	ND
FL	3.74	ND	8.14	0.73	ND	5.04	3.02	1.40	13.0	22.9	16.9	28.3	21.9	ND	8.14	0.51	2.8	14.7
PHE	13.0	ND	32.0	ND	ND	31.4	60.4	ND	1.00	88.8	ND	67.8	102	ND	32.0	5.52	15.4	69.9
AN	ND	ND	ND	ND	ND	ND	ND	ND	ND	ND	ND	ND	ND	ND	ND	ND	ND	ND
FA	2.64	2.45	ND	ND	1.06	0.71	1.23	8.09	5.55	ND	3.98	6.73	N.D	5.24	1.19	7.72	3.91	ND
PY	1.89	3.52	ND	ND	ND	1.66	1.46	4.05	2.39	ND	3.61	2.15	0.06	3.81	2.19	5.88	5.09	0.13
BaA	1.59	2.31	ND	ND	ND	ND	ND	ND	ND	ND	ND	ND	ND	1.79	ND	6.22	ND	ND
CHR	0.41	1.53	ND	ND	ND	ND	ND	ND	ND	ND	ND	ND	ND	0.7	ND	4.19	ND	0.12
BbFA	7.27	1.87	ND	ND	ND	ND	ND	ND	ND	ND	ND	ND	ND	3.32	ND	5.58	ND	ND
BkFA	ND	1.79	ND	ND	ND	ND	ND	ND	ND	ND	ND	ND	ND	ND	ND	3.24	ND	ND
BaP	16.5	8.47	ND	ND	ND	ND	ND	6.96	5.57	6.26	ND	ND	ND	11.1	ND	9.04	ND	2.8
IP	ND	3.24	ND	ND	ND	ND	ND	ND	ND	ND	ND	ND	ND	0.09	ND	1.29	ND	ND
DBahA	ND	3.88	ND	ND	ND	ND	ND	ND	ND	ND	ND	ND	ND	ND	ND	0.31	ND	ND
BghiP	0.07	1.73	ND	ND	ND	ND	ND	ND	ND	ND	ND	ND	ND	0.09	ND	0.75	ND	ND
∑PAH	47.2	30.8	53.1	12.8	1.06	43.1	94.9	23.0	27.6	143.	24.5	287.5	170.2	29.2	56.4	55.1	28.3	149.3

ND: not detected.
